# The Nature of the (Oligo/Hetero)Arene Linker Connecting Two Triarylborane Cations Controls Fluorimetric and Circular Dichroism Sensing of Various ds-DNAs and ds-RNAs

**DOI:** 10.3390/molecules28114348

**Published:** 2023-05-25

**Authors:** Lidija-Marija Tumir, Dijana Pavlović Saftić, Ivo Crnolatac, Željka Ban, Matea Maslać, Stefanie Griesbeck, Todd B. Marder, Ivo Piantanida

**Affiliations:** 1 Division of Organic Chemistry and Biochemistry, Ruđer Bošković Institute, 10000 Zagreb, Croatia; tumir@irb.hr (L.-M.T.); dijana.saftic@irb.hr (D.P.S.); ivo.crnolatac@irb.hr (I.C.); zeljka.ban@irb.hr (Ž.B.); matea.maslac@gmail.com (M.M.); 2 Institut für Anorganische Chemie and Institute for Sustainable Chemistry & Catalysis with Boron, Julius-Maximilians-Universität Würzburg, 97074 Würzburg, Germany; stefanie.griesbeck@gmx.de

**Keywords:** triarylborane, fluorescent probe, circular dichroism, DNA recognition, RNA recognition

## Abstract

A series of tetracationic bis-triarylborane dyes, differing in the aromatic linker connecting two dicationic triarylborane moieties, showed very high submicromolar affinities toward ds-DNA and ds-RNA. The linker strongly influenced the emissive properties of triarylborane cations and controlled the fluorimetric response of dyes. The fluorene-analog shows the most selective fluorescence response between AT-DNA, GC-DNA, and AU-RNA, the pyrene-analog’s emission is non-selectively enhanced by all DNA/RNA, and the dithienyl-diketopyrrolopyrrole analog’s emission is strongly quenched upon DNA/RNA binding. The emission properties of the biphenyl-analog were not applicable, but the compound showed specific induced circular dichroism (ICD) signals only for AT-sequence-containing ds-DNAs, whereas the pyrene-analog ICD signals were specific for AT-DNA with respect to GC-DNA, and also recognized AU-RNA by giving a different ICD pattern from that observed upon interaction with AT-DNA. The fluorene- and dithienyl-diketopyrrolopyrrole analogs were ICD-signal silent. Thus, fine-tuning of the aromatic linker properties connecting two triarylborane dications can be used for the dual sensing (fluorimetric and CD) of various ds-DNA/RNA secondary structures, depending on the steric properties of the DNA/RNA grooves.

## 1. Introduction

DNA and RNA are biomolecules that are essential for life, including the growth, operation, and reproduction of living organisms. These biomolecules are thus promising pharmaceutical targets for small molecules, which can bind to them, influence their biological properties, and signal binding events by a specific response. An important task is the design of small molecular sensors which are able to non-covalently bind selectively and recognize certain structures, i.e., discriminate among proteins, DNA and RNA polynucleotides, certain sequences (e.g., AT or GC), specific structural motifs, etc. [1]. Different spectroscopic signals from a small molecule probe upon binding to different biomolecules and/or different structural motifs is a very desirable characteristic, as it allows the observation of different processes and organelles within a cell.

Bis-triarylborane derivatives proved to be suitable fluorophores [2,3,4] for cell imaging [5,6,7,8,9], whereby the linker connecting the strongly π-accepting triarylborane units affects the photophysical properties of the fluorophores, type, and number of non-covalent interactions of the dye with biomolecules and, consequently, its affinity and selective recognition of specific structures.

**Scheme 1:**
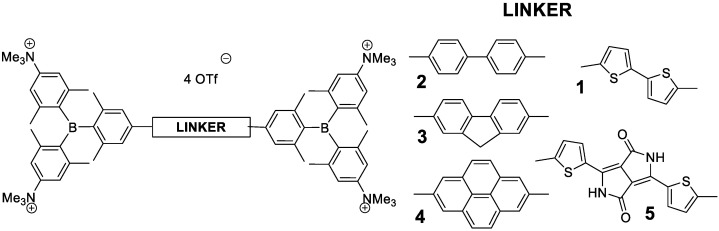
Schematic presentation of compounds **2**–**5** and, for reference, the previously studied analog **1 [10]**.

We recently demonstrated that the linker connecting two triarylborane moieties in tetracationic bis-triarylboranes has a profound impact on its fluorescence response. For example, the bis-thiophene analog (**1, Scheme 1**) showed a high emission increase, which was selective between DNA/RNA and protein [10], while the emission of its analog with 1,3-butadiyne linker (introduced as Raman-probe) [11,12] was strongly quenched by any DNA/RNA/protein. The opposite emission responses clearly demonstrated the impact of the linker on the emission properties of this type of bis-triarylborane fluorophore. Thus, to test the impact of the linker in more detail, we combined Raman-responsive chromophores with fluorophores within the same linker, which additionally demonstrated the impact of the linker on the emission response to DNA/RNA binding by introducing an aggregation-induced emission response [13]. Bis(triarylborane) tetracations linked by diethynylanthracene- and diethynyl-bis-thiophene bridges showed increased antiviral activity, a very strong and quick rise in cytotoxicity, and a very significant increase in reactive oxygen species (ROS) activity upon visible light (400–700 nm) irradiation [14].

In a previous paper [15], we demonstrated that the emission color of compounds **2**–**5** could be tuned from blue to pink by changing the linker, nicely covering the complete range of the visible spectrum. In addition, experiments on cell lines showed that all compounds very efficiently enter living cells, accumulating mostly in lysosomes, and having a negligible impact on cell proliferation, thus behaving as promising new fluorescent probes. The most intriguing compound, **5**, emits in the red region and has a large two-photon absorption cross-section. However, such promising results in intracellular sensing led to the question of what the target of our small molecules is not a trivial question, as till now, we demonstrated that close analogs (**1**) bind with similar affinity to DNA, RNA, and proteins, but, fortunately, due to its different emission properties, we attributed a protein-like target in cells rather than DNA/RNA.

To study in detail the relation between linker properties and DNA/RNA/protein binding interactions, we decided to vary steric and structural properties systematically of the linear bis-aryl-linker (Scheme 1), starting from biphenyl (**2**), characterized by high rotational freedom around the C-C single bond, then introducing rotationally “locked” fluorene (**3**) and, finally, the larger, planar pyrene (**4**) moiety. In addition, also we studied an extended version of bis-thiophene analog (**1**) by inserting a diketopyrrolopyrrole unit between two thiophenes (**5**) to see whether elongation, the high rotational flexibility and possibility of additional H-bonding of the linker within DNA/RNA grooves would have an impact on binding and sensing of various DNAs/RNAs.

## 2. Results and Discussion

**Figure 1:**
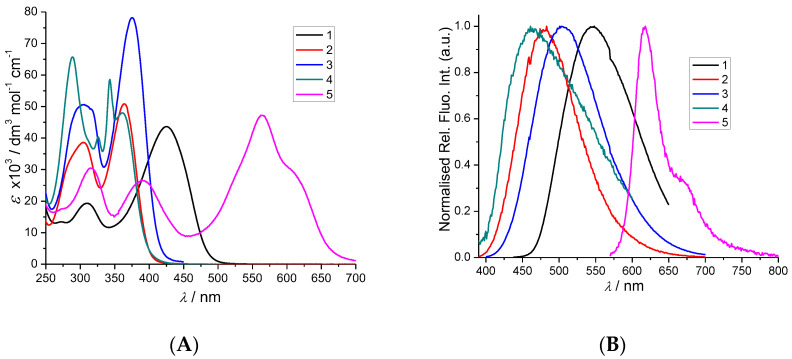
UV/vis (**A**) and fluorescence spectra ((**B**) λ_exc_ at abs. maxima; normalized to the emission maximum) of **1**–**5**. Done at pH = 7.0, buffer sodium cacodylate, *I* = 0.05 M.

Compounds **2**–**5** were prepared as described previously, and their spectrophotometric properties were previously characterized in various solvents (Supplementary Information, Table S2) [10,15]. For the purpose of the current study, UV/vis and fluorimetric spectra of **2**–**5** were collected at pH 7, in sodium cacodylate buffer (*I* = 0.05 M), in general, all spectral properties agreed well with spectra in our previous studies (Figure 1, Supplementary Information, Figures S1–S8).

More detailed analysis revealed that basic linker-modification for biphenyl (**2**)—fluorene (**3**)—pyrene (**4**) did not have a significant impact on the light absorption properties of the compounds (Figure 1A), in contrast to the introduction of the bis-thiophene linker (**1**), which red-shifted the absorbance ca. 70 nm and, in particular, the dithienyl-diketopyrrolopyrrole (**5**) induced a bathochromic shift of over 200 nm. However, the emission properties were more sensitive to the linker (Figure 1B), with pyrene analog **4** showing the smallest Stokes shift and emitting at the shortest wavelength.

All compounds showed stable fluorescence spectra over a long period, with the exception of biphenyl-analog **2**, although UV/Vis spectrum at the corresponding conditions was unchanged. For that reason, we used UV/Vis spectroscopy to study the interactions of **23** with DNA/RNA.

### 2.1. Interactions with ds-DNA and ds-RNA

#### 2.1.1. Thermal Denaturation of ds-Polynucleotides

Thermally induced dissociation of the ds-polynucleotides occurs at a well-defined temperature (*T*_m_ value), thus being used for the characterization of various ds-DNA or ds-RNA-related processes. For example, non-covalent binding of small molecules to ds-polynucleotides usually increases the thermal stability of the ds-helices, and this increase (Δ*T*_m_ value) can be correlated with the various binding modes [16] and corroborated with other independent methods.

We tested the impact of **2**–**5** on the thermal stability of various ds-DNA and ds-RNA (Supplementary Information, Figures S24–S26), and the results are summarised in Table 1. Most compounds similarly stabilized all ds-DNA/RNA, the only exception being bulky linker-analog **5**, for which stabilization of DNA/RNA was significantly weaker, likely due to the hindered insertion into DNA/RNA grooves caused by the more sterically demanding linker.

#### 2.1.2. Spectrophotometric Titrations of **2**–**5** with ds-DNA/RNA

**Figure 2:**
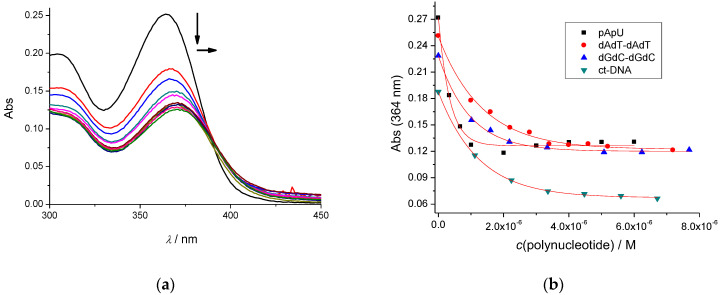
(**a**) The UV/vis titration of **2** (*c* = 1 × 10^−6^ M) with poly(dAdT)_2_; (**b**) dependence of absorption at λ_max_= 364 nm on *c*(polynucleotide), the red line is non-linear least square fitting of experimental data to Scatchard eq. (McGhee, von Hippel formalism) [17,18]. Done at pH 7, sodium cacodylate buffer, *I* = 0.05 M.

The addition of DNA/RNA caused significant changes in the UV/Vis spectra of all compounds, characterized by hypochromic and bathochromic effects (Figure 2 and Supplementary Information, Figures S9–S12). However, in most cases, changes in the UV/Vis spectra were observed up to the point of saturation of the binding sites on ds-DNA/RNA (ratio r_[dye]/[polynucleotide]_ ~ 0.2), suggesting very high affinities. Such changes do not allow accurate processing of the titration data by non-linear fitting procedures adapted from the Scatchard equation [17,18], which are used for the calculation of binding constants. The exception was biphenyl-derivative **2**, allowing an estimation of the binding constants (Figure 2a, Table 2).

To circumvent this problem, we took advantage of the strong fluorescence of **3**–**5**, enabling fluorimetric titrations at several orders of magnitude lower concentrations of the dyes, thus allowing the collection of at least part of the titration data at an excess of DNA/RNA over dye. Detailed comparison of fluorimetric titrations (Figure 3, Figure 4 and Figure 5; Table 2, Supplementary Information, Figures S13–S23) revealed that the emission response was strongly affected by the linker, as well as by the secondary structure of the ds-polynucleotide.

**Figure 3:**
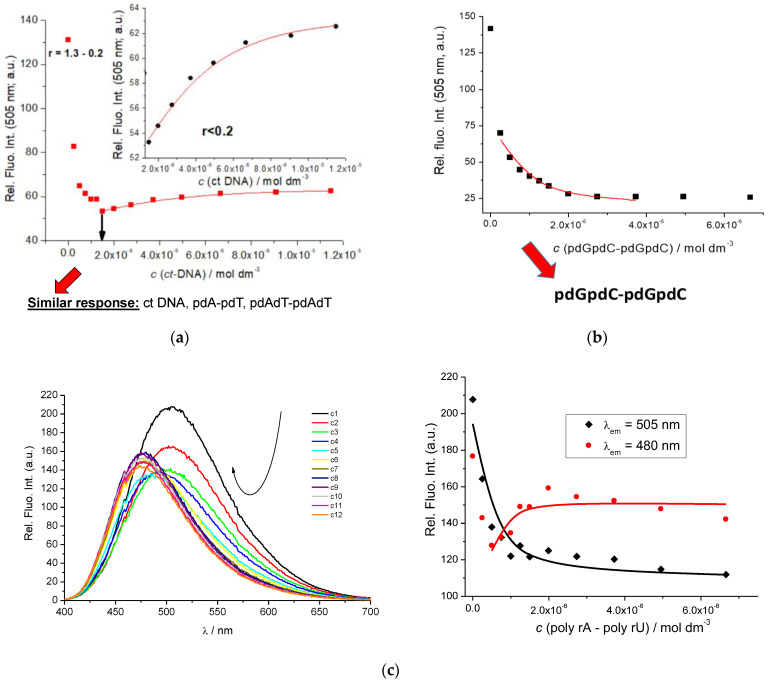
Fluorimetric titration of **3**, λ_exc_ = 375 nm, λ_em_ = 505 nm, *c* = 2 × 10^−7^ M with (**a**) ct-DNA, AT-DNAs; (**b**) poly dGdC-poly dGdC; (**c**) poly rA—poly rU; Experimental (■ or ■) and calculated (― or ―) fluorescence intensities of **3** upon addition of RNA. Done at pH = 7.0, Na cacodylate buffer, *I* = 0.05 M.

Particularly intriguing was the fluorimetric response of fluorene analog **3**, showing emission quenching only for GC-DNA (Figure 3b), whereas, for AT-containing DNAs, strong aggregation-induced quenching was followed by an emission increase at r < 0.2 (Figure 3a). Such selectivity in response can be correlated to the steric properties of the DNA minor groove (Supplementary Information Table S1), whereby the AT-DNA groove is very efficient for small molecule binding (thus, all groove binders bind to AT-DNA [19]) and also supports aggregation at an excess of a dye over DNA [20] (r > 0.2, emission quenching), whereas sterically hindered GC-DNA does not support aggregation but only loose binding of single molecules—thus allowing a single binding mode. As among ds-DNA bases, guanine is the easiest nucleobase to oxidize [21]; it commonly induces pronounced emission quenching [22], at variance to AT-sequences which can induce emission increase.

An even more intriguing response of **3** was observed for the AU-RNA exclusively (Figure 3c). Thus, a strong hypsochromic shift (−25 nm) of the emission maximum was observed, whereas the relative intensity of emission changed only negligibly. The selective response of **3** could be attributed to the ds-RNA secondary structure (see Supplementary Information Table S1 for structural details), characterized by a very deep and narrow major groove, which can accommodate small molecules differently from the minor groove of DNAs, consequently resulting in the finely tuned fluorimetric response of **3**.

**Figure 4:**
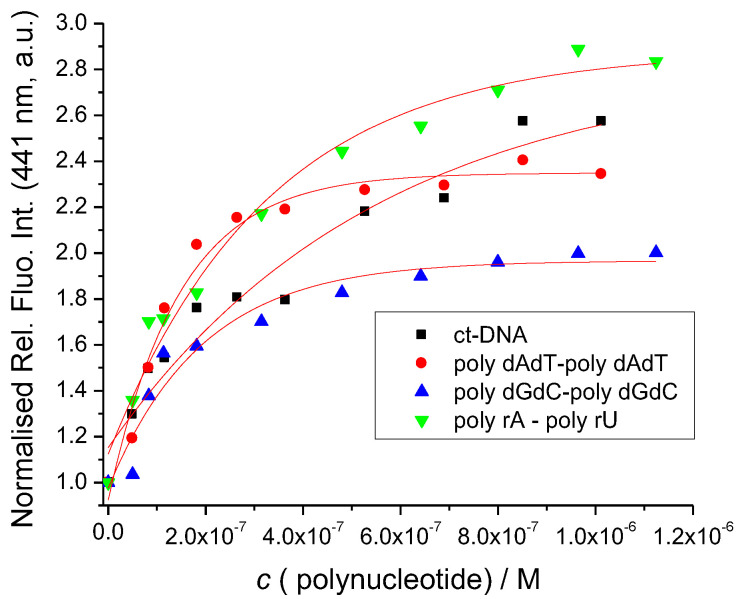
Fluorimetric titration of **4**, λ_exc_ = 345 nm, *c* = 5.0 × 10^−8^ M with polynucleotides; experimental (●, ▲, ■, ▲) and fit the Scatchard eq. (―) fluorescence intensities at λ_em_ = 441 nm. Done at pH = 7.0, Na cacodylate buffer, *I* = 0.05 M.

In comparison to fluorene (**3**), further increase of rigidity and the aromatic surface in pyrene-analog (**4**) resulted in a complete loss of selectivity; its emission change was exclusively enhanced by any ds-DNA/RNA studied (Figure 4).

**Figure 5:**
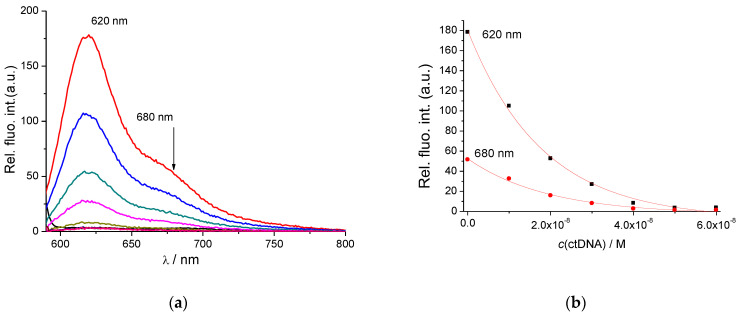
(**a**) Changes in the fluorescence spectrum of **5** (*c* = 5.0 × 10^−8^ M, λ_exc_ = 562 nm) upon titration with **ctDNA**; (**b**) Dependence of **5** emissions at λ_max_ = 620 nm and λ = 680 nm experimental (■, ●) and fit the Scatchard eq. (―) fluorescence intensities on c(ctDNA), at pH 7, sodium cacodylate buffer, *I* = 0.05 M.

Intriguingly, whereas previously studied very flexible bis-thiophene-linker (**1**) showed only emission increase for any DNA/RNA added [10], the insertion of diketopyrrolopyrrole between two thiophenes (**5**) completely reversed the response, showing exclusively fluorescence quenching for any of ds-DNA/RNA (Figure 5). 

Correlation of the emission responses to linker properties of **1**, **3**–**5** revealed that neither length nor rigidity/planarity of the linker controls the emission of the complexes formed. In fact, it seems that the decisive role involves a fine interplay between the binding site (DNA/RNA groove size and shape differing for each DNA/RNA studied) and the electronic properties of the compete chromophore system (including communication between the triarylborane and the linker).

Processing of fluorimetric titration data by means of the Scatchard equation (McGhee, vonHippel formalism) [17,18] yielded binding constants (Table 2), whereby somewhat lower values obtained for **2** can be attributed to the less sensitive titration method (UV/vis). The other compounds showed mostly rather similar binding affinities to all ds-DNAs/RNAs studied (log*Ks* = 7–8), pointing out that the different emission responses do not reflect differences in binding interactions.

#### 2.1.3. Circular Dichroism

So far, we have studied the non-covalent interactions at 25 °C by monitoring the spectroscopic properties of compounds **2**–**5** upon the addition of the polynucleotides. In order to obtain insight into the changes in polynucleotide properties induced by the small molecule binding, we chose CD spectroscopy as a highly sensitive method for the examination of conformational changes in the secondary structure of polynucleotides [23]. In addition, **2**–**5**, as achiral small molecules, can still generate an induced CD spectrum (ICD) upon binding to polynucleotides, which could give useful information about the modes of interaction [24,25].

The fluorene analog **3** did not show any induced (I)CD bands but only caused a decrease in the CD bands of DNA/RNA < 300 nm (Supplementary Information, Figures S29 and S30). Compound **5** showed only a very weak negative induced (I)CD band at 315 nm for GC-DNA, and for other DNAs/RNAs, the addition of **5** only caused a decrease in the CD bands of DNA/RNA < 300 nm (Supplementary Information, Figures S32 and S33). Such a response is in accord with previously studied compound **1**, as well as its analog with the 1,3-butadiyne linker [11], attributed to the binding of a dye to DNA/RNA grooves (thus only slightly affecting the helicity of DNA/RNA), whereas the absence of ICD bands was a consequence of the unfavorable orientation of the dye transition dipole moments with respect to the DNA/RNA chiral axis. Namely, if the dominant transition dipole moment of the dye is coplanar with the base pairs, it will yield a weak negative ICD, whereas if it is at a ca. 45° angle to the DNA chiral axis, it will give a strong positive ICD [24,25]; however, if is at an angle in-between, the positive and negative components could cancel each other out.

**Figure 6:**
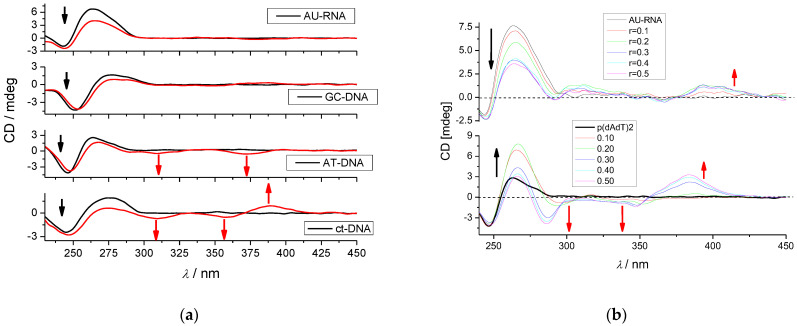
(**a**) CD titration of polynucleotides (*c* = 2 × 10^−5^ M) with **2** at molar ratio *r*_[compound]/[polynucleotide]_ = 0.3. Red arrows denote ICD bands > 300 nm, and black arrows denote the decrease of CD bands < 300 nm belonging to the DNA/RNA helix; (**b**) CD titration of AT-DNA and AU-RNA (*c* = 2 × 10^−5^ M) with 4 at molar ratios *r*_[compound]/[polynucleotide]_ = 0, 0.1, 0.2, 0.3, 0.4, and 0.5. Red arrows denote ICD bands > 300 nm, and black arrows denote a decrease of CD bands < 300 nm belonging to the DNA/RNA helix. The addition of **4** to GC-DNA or ct-DNA did not yield any ICD bands. Done at pH 7.0, buffer sodium cacodylate, *I* = 0.05 M.

However, for several DNAs/RNAs, the biphenyl analog **2** and pyrene analog **4** showed moderate or even strong ICD bands > 300 nm. For example, **2** showed ICD bands only for AT-containing DNAs but no signal > 300 nm for AU-RNA and GC-DNA (Figure 6a). Such a selective response can be attributed to the well-defined minor groove of AT-sequences (see Supplementary Information Table S1), in which rod-like bis-triarylboranes fit nicely [10,11], and, in the case of **2**, the biphenyl-linker orients uniformly in respect to the AT-DNA helical axis, thus producing ICD bands [24,25].

For pyrene analog **4**, the selectivity of the ICD band > 300 nm response is even more pronounced. The strong positive ICD bands at >350 nm obtained for AU-RNA and AT-DNA are similar (Figure 6b), suggesting that the pyrene-linker is uniformly oriented in the grooves of narrow width at an angle of ca. 45° with respect to the polynucleotide chiral axis [24,25] (Supplementary Information Table S1; see RNA major groove and AT-DNA minor groove), whereas the amino group of guanine in GC-DNA sterically hinders insertion of pyrene and, thus, completely abolished any ICD band > 300 nm (Supplementary Information Figure S31). Furthermore, in the 230–300 nm range (where intrinsic CD bands of DNA/RNA appear), there is a clear difference between the **4**/AU-RNA and **4**/AT-DNA CD spectra, which can be mostly attributed to strong differences in secondary structure between the A-helix of RNA and B-helix of DNA, as well as a resulting different orientation of bound **4** within the corresponding binding sites.

Comparison of all CD data reveals that fine-tuning of linker-chromophore transition dipole moments with respect to the DNA/RNA chiral axis can control selective ICD response to a particular type of DNA or RNA binding site.

## 3. Materials and Methods

### 3.1. General Procedures

UV-Vis absorption spectra of compounds, UV-Vis titrations, and thermal melting experiments were measured on a Varian Cary 100 Bio spectrometer. Fluorescence spectra were recorded on a Varian Cary Eclipse fluorimeter. CD spectra were recorded on the JASCO J815 spectrophotometer. UV-Vis, fluorescence, and CD spectra were recorded using 1 cm path quartz cuvettes. Polynucleotides were purchased as noted: calf thymus (*ct*)-DNA, poly dAdT—poly dAdT, poly dGdC—poly dGdC, and poly rA—poly rU (Sigma) and dissolved in sodium cacodylate buffer, *I* = 0.05 M, pH = 7.0. The calf thymus (*ct*-) DNA was additionally sonicated and filtered through a 0.45 μm filter [26]. Polynucleotide concentration was determined spectroscopically and expressed as the concentration of phosphates [27]. Stock solutions of compounds **2**–**5** were prepared by dissolving the compounds in H_2_O or DMSO; total DMSO content was below 1% in UV-Vis and below 0.1% in fluorimetric measurements. All measurements were performed in sodium cacodylate buffer, *I* = 0.05 M, pH = 7.0. Concentrations of **2**–**5** below 2 × 10^−5^ M were used for UV-Vis absorbance measurements to avoid intermolecular association.

### 3.2. UV/Vis, CD, and Fluorescence Titrations

UV-Vis and fluorimetric titrations were performed by adding portions of polynucleotide solution into the solution of the compound studied. After mixing polynucleotides with the compounds, it was observed that equilibrium was reached in less than 120 s. UV-Vis titrations were measured in the wavelength range of 250–450 nm. In fluorimetric titrations, the concentrations of **2**–**5** were 5 × 10^−8^–2 × 10^−7^ M. The excitation wavelength of λ_exc_ > 350 nm was used to avoid absorption of excitation light caused by increasing absorbance of the polynucleotide. Titration data obtained for ds-DNA and ds-RNA were fitted by the non-linear least square method (using Origin 7.0 software) to the McGhee, vonHippel formalism of Scatchard equation [17,18], whereby simultaneously binding constant (logKs) and Scatchard ratio n_[bound molecule/DNA]_ were calculated. Calculations mostly gave values of ratio n = 0.2 ± 0.05, but for easier comparison, all *Ks* values were re-calculated for a fixed n = 0.2. Values for *Ks* have satisfactory correlation coefficients (>0.98).

CD experiments were performed by adding portions of stock solutions of compound **2**–**5** into a solution of the polynucleotides (c ≈ 1–2 × 10^−5^ M). Compounds **2**–**5** are achiral and, therefore, do not possess intrinsic CD spectra. CD spectra were recorded with a scan speed of 200 nm/min. Buffer background was subtracted from each spectrum, while each spectrum was the result of three accumulations.

### 3.3. Thermal Denaturation Experiments

Thermal denaturation curves for ds-DNA, ds-RNA, and their complexes with compounds **2**–**5** were determined by following the absorption change at 260 nm as a function of temperature [16]. The absorbance scale was normalized. *T_m_* values are the midpoints of the transition curves determined from the maximum of the first derivative and checked graphically by the tangent method [28]. The Δ*T_m_* values were calculated by subtracting *Tm* of the free nucleic acid from the *T_m_* of the complex. Every Δ*T_m_* value reported herein was the average of at least two measurements. The error in Δ*T_m_* is ±0.5 °C.

## 4. Conclusions

We studied how DNA and RNA interacted with tetracationic quadrupolar chromophores, which include three-coordinate triarylboron π-acceptors that were connected by various π-linkers: biphenyl (**2**), fluorine (**3**), pyrene (**4**), and dithienyl-diketopyrrolopyrrole (**5**). Our primary objective was to investigate the effect of the different linkers joining two triarylborane units on their binding to DNA/RNA. It was previously found that the compounds enter the HeLa cells and localize at the lysosomes without any effect on cell viability [15]. However, regarding their use in bioimaging, only compounds **1**, **3**, and **5** absorb in the visible range (>390 nm), optimal for bioimaging, whereas others require either UV excitation or two-photon absorption in the NIR.

All compounds very strongly bind to all ds-DNAs or ds-RNAs studied, thus again stressing the general applicability of our bis-triarylborane dications as newly developed dyes for ds-DNA/RNA sensing. The binding sites of the dyes are the ds-DNA minor groove or ds-RNA major groove.

The relation between the fluorimetric response of the dyes upon DNA/RNA binding and the structure of the dye or the secondary structure of the DNA/RNA was quite complex. The electronic and steric characteristics of the chromophore systems (triarylborane moieties and the π-linkers between them) and binding site (DNA/RNA groove size and shape varying for each DNA/RNA investigated, Table S1) influenced each other, so their interactions yield redistribution of the chromophore dipole moments and fine conformation tuning, that finally yield the particular fluorescence responses.

The results presented herein show that large and rigid aromatic linkers (**4**, **5**) give similar emission response to all DNAs/RNAs studied, whereas somewhat smaller linker, like fluorene **3**, has some adaptability to the particular structure of the binding site of different ds-DNA or ds-RNA, which results in fluorimetric recognition between AT-DNAs, GC-DNA, and AU-RNA.

Circular dichroism (CD) spectroscopy, as a structurally sensitive method, demonstrated that all dyes studied cause a minor decrease in DNA/RNA helicity, in line with a groove binding site. However, induced (I)CD bands were observed in the 300–400 nm range only for some dyes, with no apparent relation to the linker rigidity or steric volume. For instance, **3** (fluorene linker) and **5** (dithienyl-diketopyrrolopyrrole linker) yielded only negligible induced CD signals, suggesting the unfavorable orientation of the dye transition dipole moments with respect to the DNA/RNA chiral axis. Oppositely, the addition of **2** (biphenyl linker) yielded pronounced ICD signals specifically upon binding to DNA containing AT-sequences as the consequence of a tight fit in the AT-DNA minor groove. Compound **4**, with a much larger, planar aromatic surface linker (pyrene), also gave a specific ICD band for AT-DNA but not for GC-DNA. However, at variance to the flexible biphenyl-**2**, pyrene analog **4** also yielded a strong ICD band for AU-RNA, pointing out a tight and well-oriented fit within the ds-RNA major groove.

This study shows that systematic variation of the properties of linkers between triarylborane π-acceptors (flexibility vs. rigidity, aromatic surface size, and participation of nitrogen/sulfur heteroatoms) can have a strong impact on the fluorimetric and circular dichroism recognition of various ds-DNA/RNA secondary structures, depending on the properties of the DNA/RNA grooves. Obtained results are in line with complementary advances in the field of strongly sensitive fluorimetric probes for biomacromolecules [29,30,31].

## Figures and Tables

**Table 1:** The ^a^ Δ*T*_m_ values (°C) of studied ds-polynucleotides upon addition of ratio ^b^ *r* = 0.1 of **1**–**5** at pH = 7.0 (sodium cacodylate buffer, *I* = 0.05 M).

	ct-DNA	p(dAdT)_2_	poly rA—poly rU
**^c^ 1**	7.3	10.0	9.5
**2**	4.5	6.7	12.1
**3**	10.0	8.2	9.3
**4**	8.0	9.6	7.4
**5**	3.4	-	0.6

^a^ Error in Δ*T*_m_: ±0.5 °C; ^b^ *r* = [compound]/[polynucleotide], ^c^ Previously published results [10].

**Table 2:** Binding constants (log*Ks*) and spectroscopic properties of **1**–**5** with polynucleotides calculated by processing fluorimetric ^a^ or ^b^ UV/vis titrations.

	1 [10]	2	3	4	5
ct-DNA	7.0	^b ^ 6.1	6.9	7.1	7.7
p(dAdT)_2_	7.9	^b ^ 6.1	6.9	8.7	7.7
p(dGdC)_2_	7.6	^b ^ 6.0	7.4	8.0	7.9
prAprU	^b^ 7	^b ^ 7.5	^c^ 7.9	7.4	7.7

^a^ Calculated using the Scatchard eq. [17,18] for a fixed n = 0.2; colors denote INCREASE or QUENCHING of fluorescence emission at an excess of DNA/RNA over dye. ^b ^UV/Vis titrations. ^c^ Strong hypsochromic shift of the emission maximum caused quenching at starting λ_max_ and an increase at the new λ_max_.

## Data Availability

Not applicable.

## Supplementary Materials

The following supporting information can be downloaded at: https://www.mdpi.com/article/10.3390/molecules28114348/s1. References [15,32,33] are cited in the Supplementary Materials.

## Sample Availability

Stock solutions of samples are available from the authors.
