# Supplementary material for: The Nature of the (Oligo/Hetero)Arene Linker Connecting Two Triarylborane Cations Controls Fluorimetric and Circular Dichroism Sensing of Various ds-DNAs and ds-RNAs
Source: Molecules. 2023 May 25;28(11):4348. doi: 10.3390/molecules28114348 (PMC10254923; doi:10.3390/molecules28114348)
Supplement: Supplementary file 1 [file molecules-28-04348-s001.zip › molecules-2407546-supplementary.pdf]

Supporting Information

The nature of the (oligo/hetero)arene linker connecting two triarylborane cations controls fluorimetric and circular dichroism sensing of various ds-DNAs and ds-RNAs

Lidija-Marija Tumir [1], Dijana Saftić Pavlović [1], Ivo Crnolatac [1], Željka Ban [1], Matea Maslać [1], Stefanie Griesbeck [2], Todd B. Marder [2,]* and Ivo Piantanida [1,]*

[1]Division of Organic Chemistry & Biochemistry, Ruđer Bošković Institute, Zagreb, Croatia.

[2]Institut für Anorganische Chemie, and Institute for Sustainable Chemistry & Catalysis with Boron, Julius-Maximilians-Universität Würzburg, Würzburg, Germany.

Table of Contents

General information
Spectrophotometric titrations
Thermal melting experiments
CD experiments
References

Table S1. Groove widths and depths for selected nucleic acid conformations[1,2].

Structure type	Groove width [Å]		Groove depth [Å]	
	major	minor	major	minor
[a] poly rA – poly rU	3.8	10.9	13.5	2.8
[b] B-DNA (e.g. ct-DNA)	11.7	5.7	8.5	7.5
[b] poly dGdC – poly dGdC	13.5	9.5	10.0	7.2
[b] poly dAdT – poly dAdT	11.2	6.3	8.5	7.5

[a] A-helical structure (e.g., A-DNA or RNA)

[b] B- helical structure (e.g., B-DNA)

Spectrophotometric data collected in buffer system:

Table S2. The photophysical data of compounds **2-5** in water [3]

	λ_{abs}/nm	ε / M^{-1} cm^2	λ_{em}/nm	Stokes shift/ cm^{-1}	Φ_f
**2**	364	57000	486	6900	0.58
**3**	375	62000	513	7200	0.33
**4**	365	61000	467	6000	0.12
**5**	594	50000	620	7100	0.13

Compound **2** 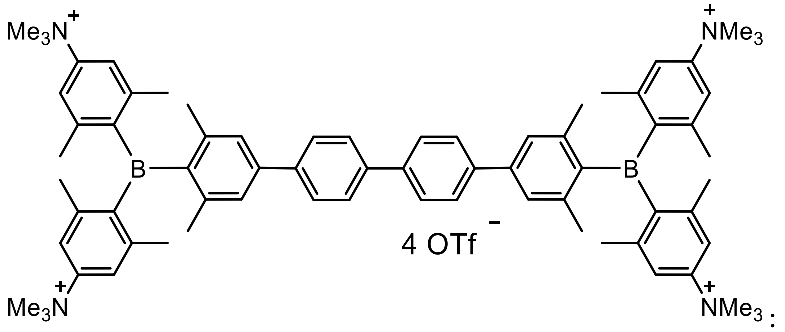

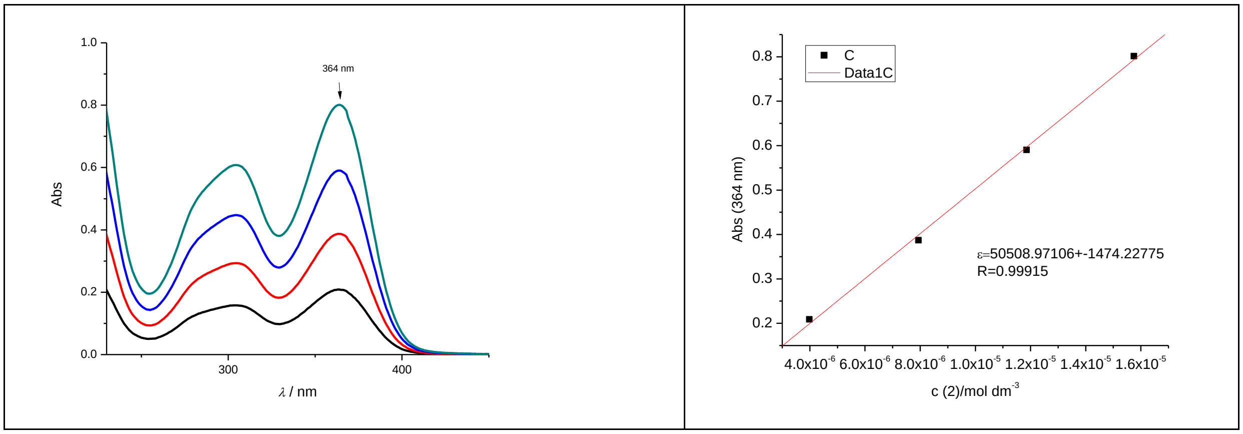

Figure S1. Left: UV/Vis spectra of **2** (concentration range from $4 \times 10^{-6} – 1.6 \times 10^{-5}$ M); Right: linear dependence of the absorbance at 364 nm on the **2** concentration (Na-cacodylate buffer, I = 0.05 M, pH = 7.0).

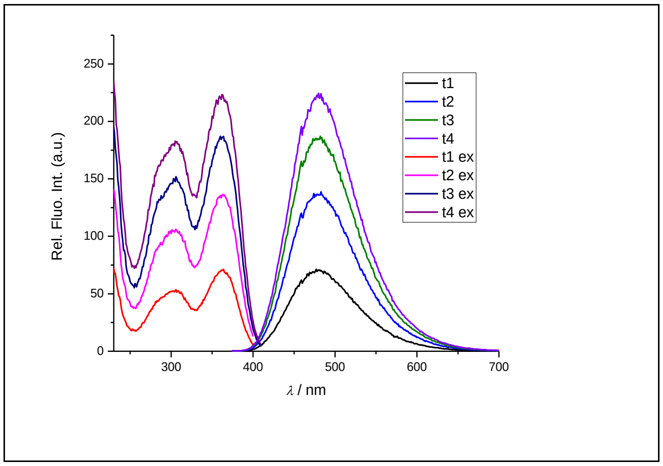

Figure S2. Excitation (λ_{em} = 480 nm) and emission (λ_{exc} = 364 nm) fluorescence spectra of **2**. (Na-cacodylate buffer, I = 0.05 M, pH = 7.0).

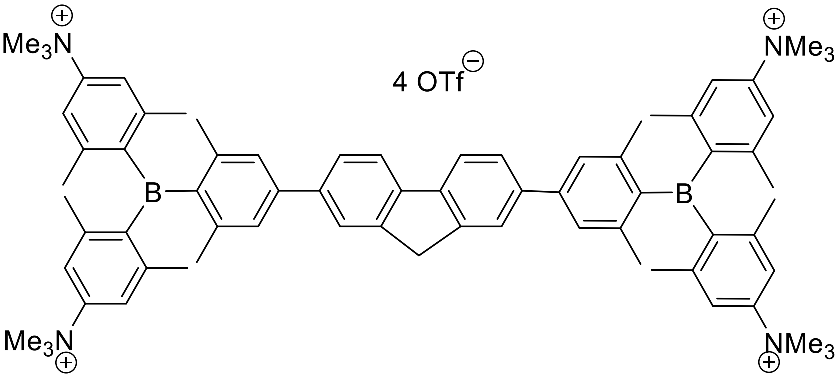

Compound **3**

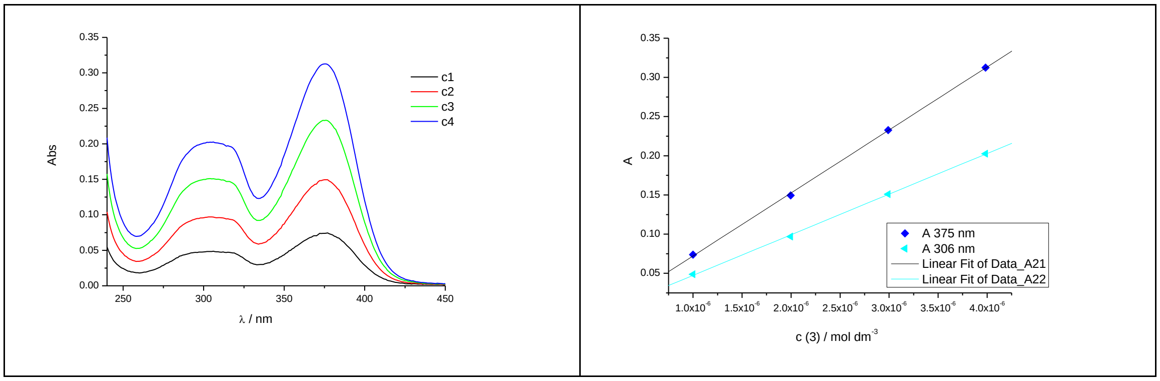

Figure S3. Left: UV/Vis spectra of **3** (concentration range from 1 – 4 × 10^{-6} M); Right: linear dependence of the absorbance at 306 and 375 nm on the **3** concentration (Na-cacodylate buffer, I = 0.05 M, pH = 7.0).

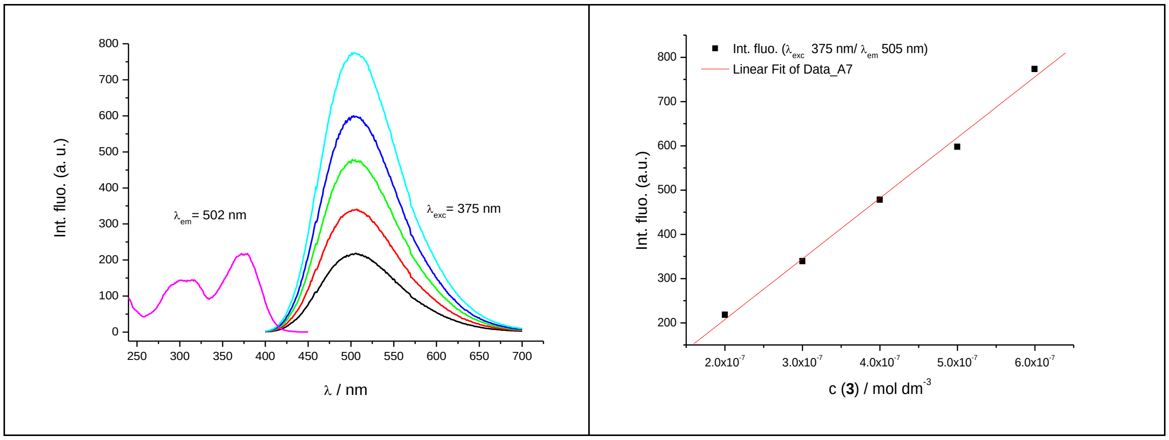

Figure S4. Left: Excitation (λ_{em} = 502 nm, c = 2 × 10^{-7} M) and emission (λ_{exc} = 375 nm) fluorescence spectra of **3** (c1-c5 = 2 – 6 × 10^{-7} M); Right: linear dependence of the fluorescence intensity on the **3** concentration (λ_{exc} = 375 nm, λ_{em} = 505 nm, Na-cacodylate buffer, *I*=0.05 M, pH = 7.0).

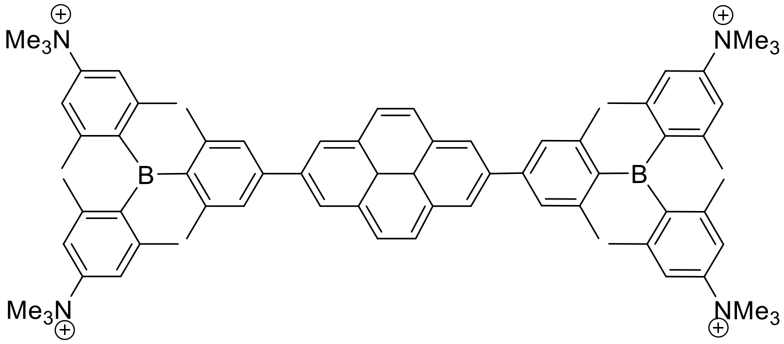

Compound **4**

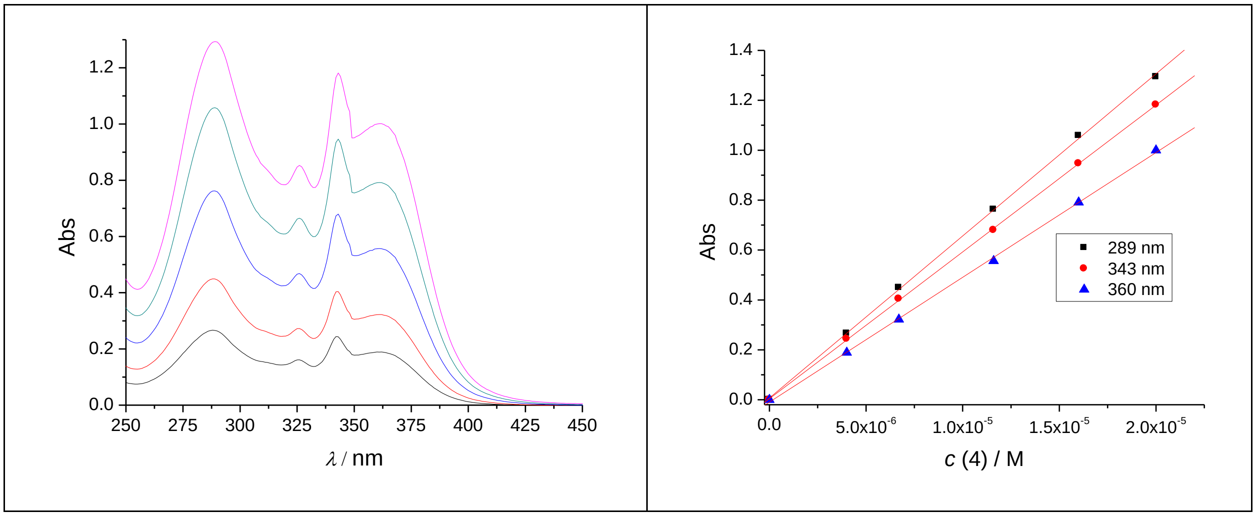

Figure S5. Left: UV/Vis spectra of **4** (concentration range from 4 – 20 × 10^{-6} M); Right: linear dependence (—) of the absorbance at different wavelengths on the **4** concentration (Na-cacodylate buffer, *I* = 0.05 M, pH = 7.0).

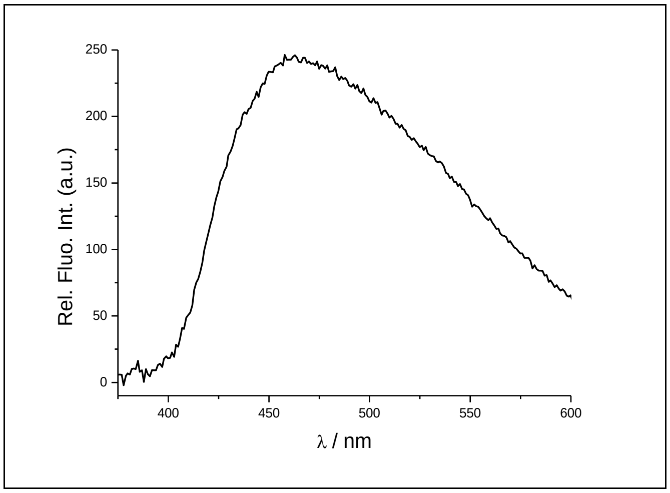

Figure S6. Emission fluorescence spectra of **4** (λ_{exc} = 345 nm, c = 5 × 10^{-8} M Na-cacodylate buffer, I = 0.05 M, pH = 7.0).

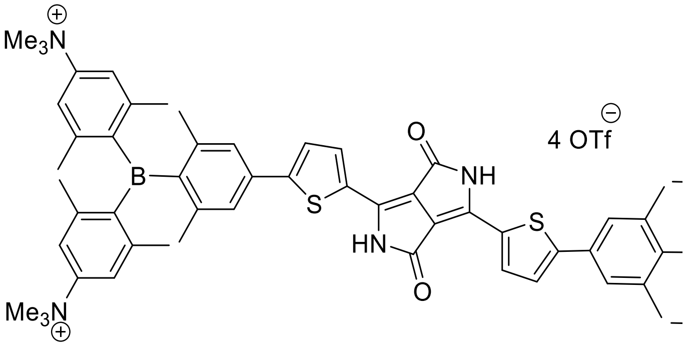

Compound **5**:

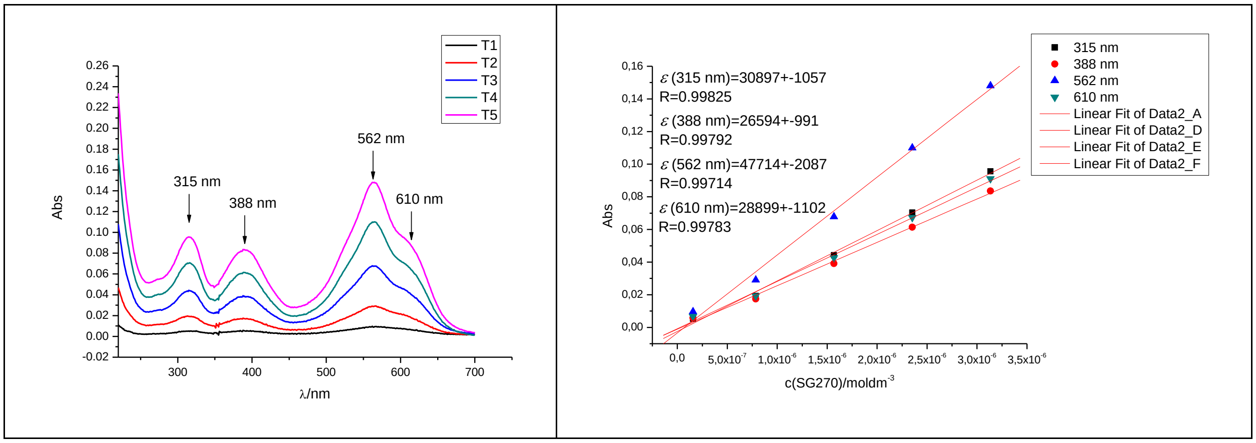

Figure S7. Left: UV/Vis spectra of **5** (concentration range from 1.6 × 10^{-7} – 3.1 × 10^{-6} M); Right: linear dependence of the absorbance different wavelengths on the **5** concentration (Na-cacodylate buffer, I = 0.05 M, pH = 7.0).

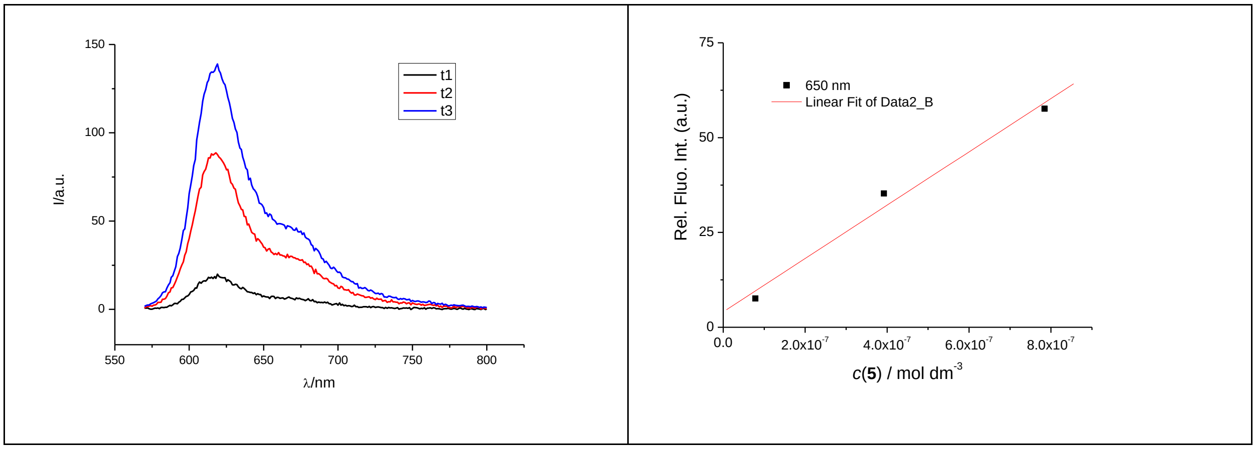

Figure S8. Left: Emission (λ_{exc} = 562 nm) fluorescence spectra of **5** ($8 \times 10^{-8} - 2.4 \times 10^{-6}$ M); Right: Dependence of the fluorescence intensity on the **5** concentration at different emission wavelengths (λ_{exc} = 562 nm, λ_{em} = 505 nm, Na-cacodylate buffer, I = 0.05 M, pH = 7.0).

Titrations with DNA/RNA:

UV/vis titrations:

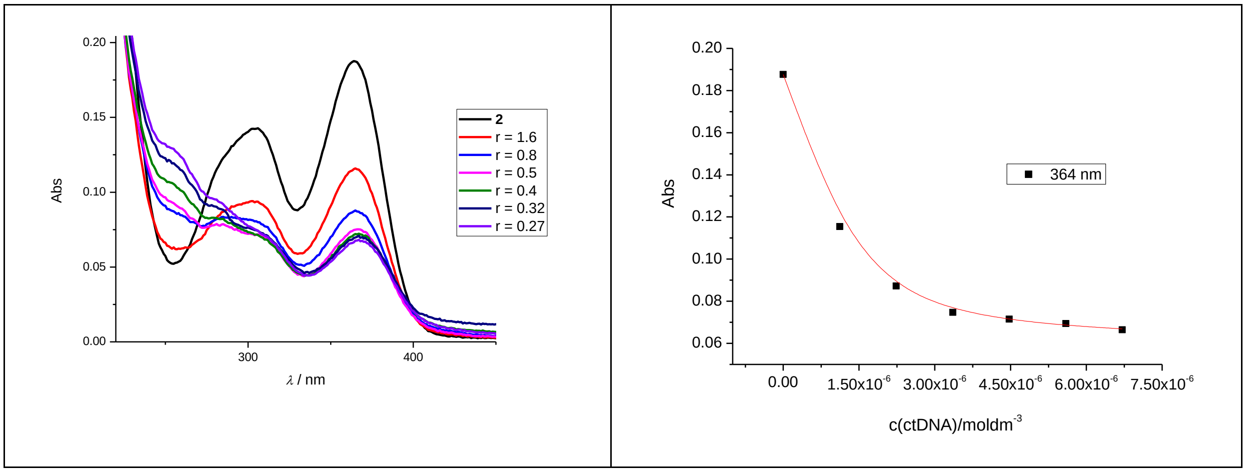

Figure S9. Left: UV-Vis titration of **2** (c = 1 × 10^{-6} mol dm^{-3}) with *ct*-DNA; Right: dependence of absorption at λ_{max} = 364 nm on c (*ct*-DNA); (Na-cacodylate buffer, I = 0.05 M, pH = 7.0)

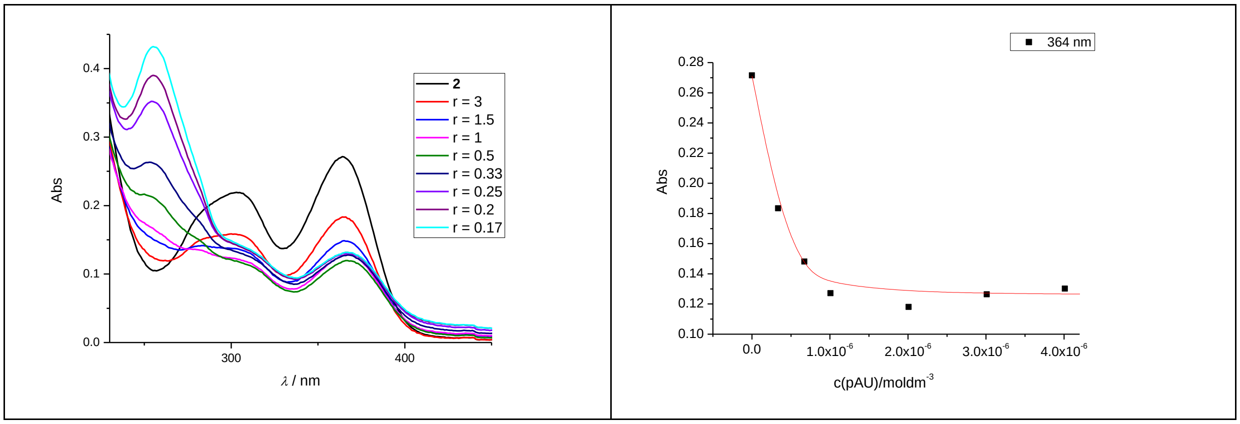

Figure S10. Left: UV-Vis titration of **2** (c = 1 × 10^{-6} mol dm^{-3}) with polyA – polyU; Right: dependence of absorption at λ_{max} = 364 nm on c (polyA – polyU); (Na-cacodylate buffer, I = 0.05 M, pH = 7.0)

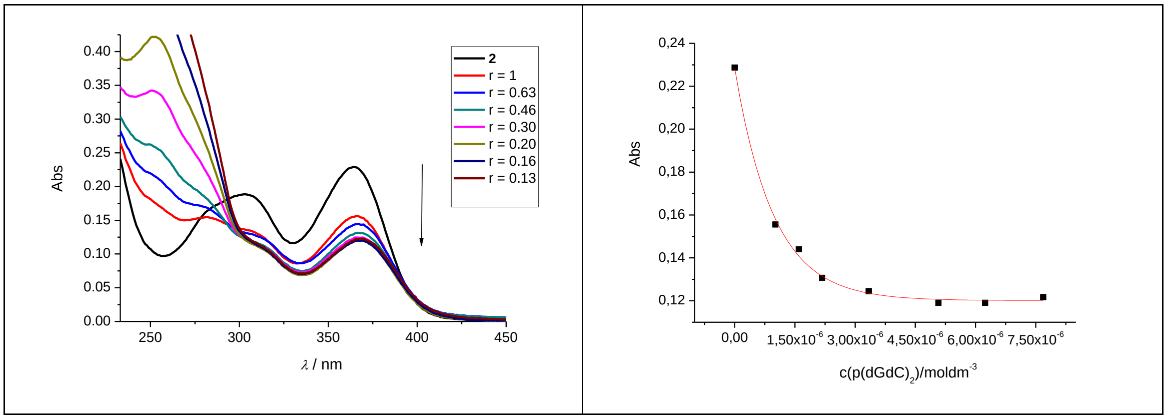

Figure S11. Left: UV-Vis titration of **2** ($c = 1\times10^{-6}$ mol dm^{-3}) with poly(dGdC)$_2$; Right: dependence of absorption at $\lambda_{max} = 364$ nm on c (poly(dGdC)$_2$); (Na-cacodylate buffer, $I = 0.05$ M, pH = 7.0)

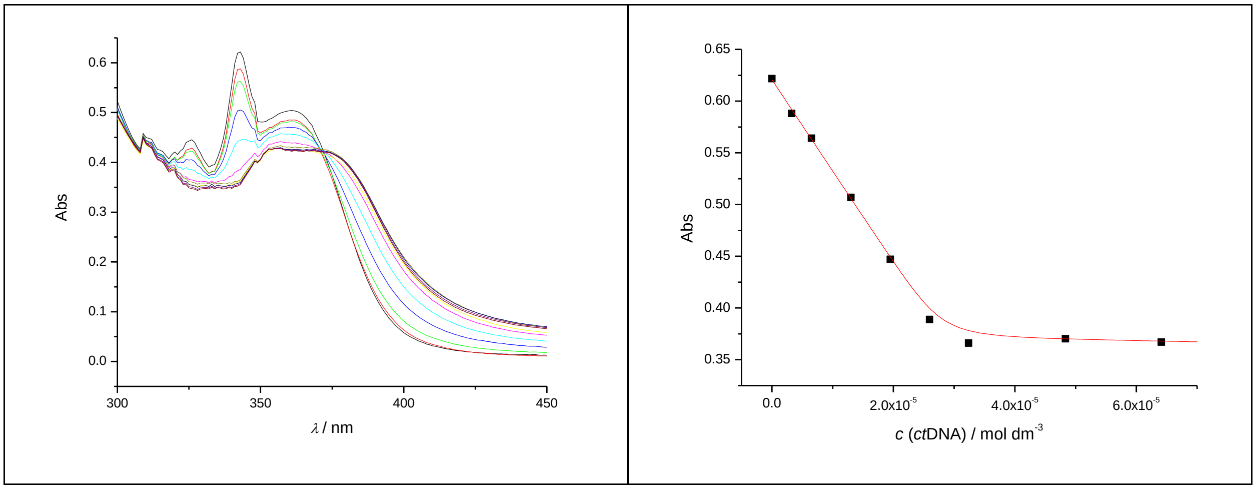

Figure S12. Left: UV-Vis titration of **4** ($c = 1\times10^{-6}$ mol dm^{-3}) with *ct*DNA; Right: dependence of absorption at $\lambda_{max} = 343$ nm on c (*ct*DNA); (Na-cacodylate buffer, $I = 0.05$ M, pH = 7.0)

Fluorimetric titrations:

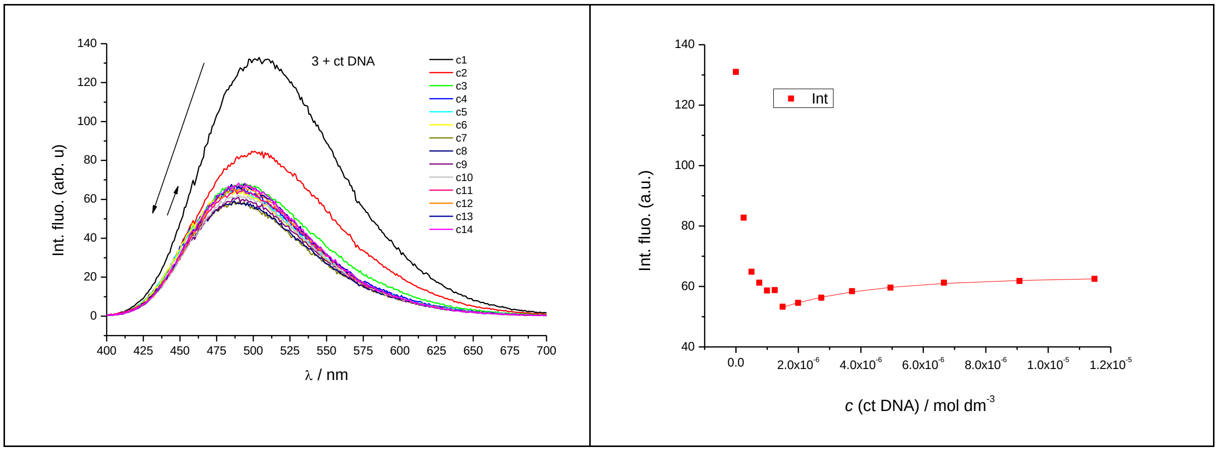

Figure S13. Left: Fluorimetric titration of **3**, λ_{exc} = 375 nm, c = 2 × 10^{-7} mol dm^{-3} with ct-DNA, Right: Experimental and calculated fluorescence intensities of **3** at λ_{em} = 505 nm upon addition of *ct*-DNA (Na-cacodylate buffer, *I* = 0.05 M, pH = 7.0)

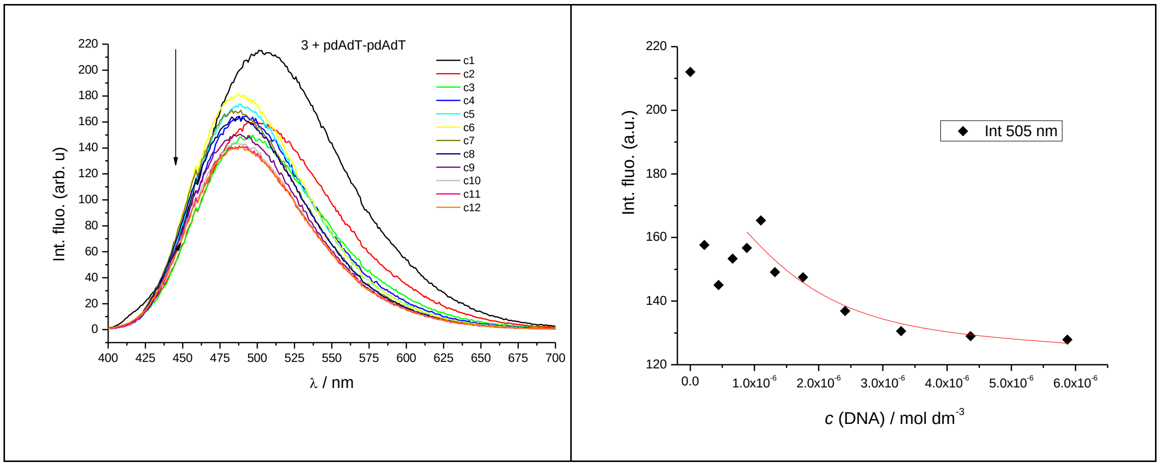

Figure S14. Left: Fluorimetric titration of **3**, λ_{exc} = 375 nm, c = 2 × 10^{-7} mol dm^{-3} with pdApdT-pdApdT, Right: Experimental and calculated fluorescence intensities of **3** at λ_{em} = 505 nm upon addition of DNA (Na-cacodylate buffer, *I* = 0.05 M, pH = 7.0).

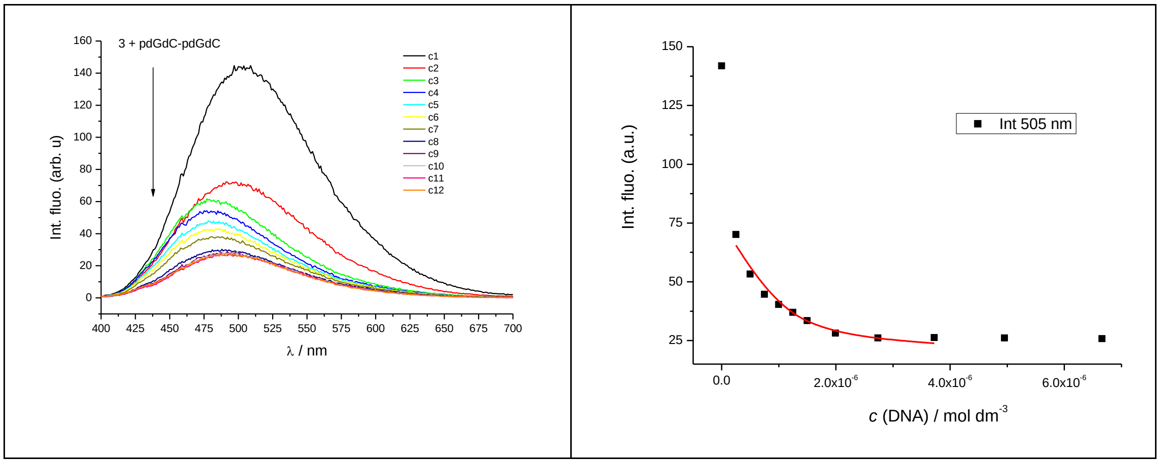

Figure S15. Left: Fluorimetric titration of **3**, λ_{exc} = 375 nm, c = 2 × 10^{-7} mol dm^{-3} with pdGpdC-pdGpdC, Right: Experimental and calculated fluorescence intensities of **3** at λ_{em} = 505 nm upon addition of DNA (Na-cacodylate buffer, *I* = 0.05 M, pH = 7.0).

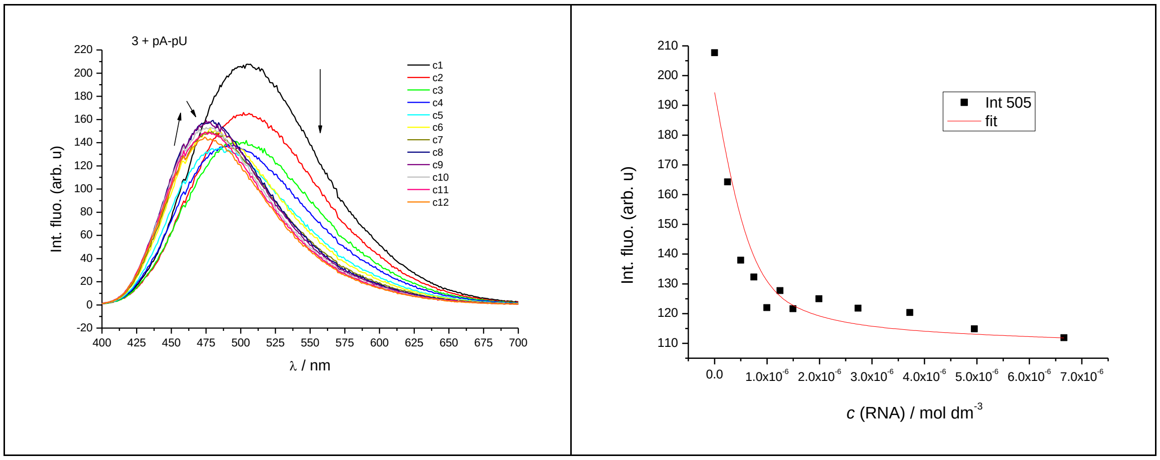

Figure S16. Left: Fluorimetric titration of **3**, λ_{exc} = 375 nm, c = 2 × 10^{-7} mol dm^{-3} with pA-pU, Right: Experimental and calculated fluorescence intensities of **3** at λ_{em} = 505 nm upon addition of DNA (Na-cacodylate buffer, *I*=0.05 M, pH = 7.0).

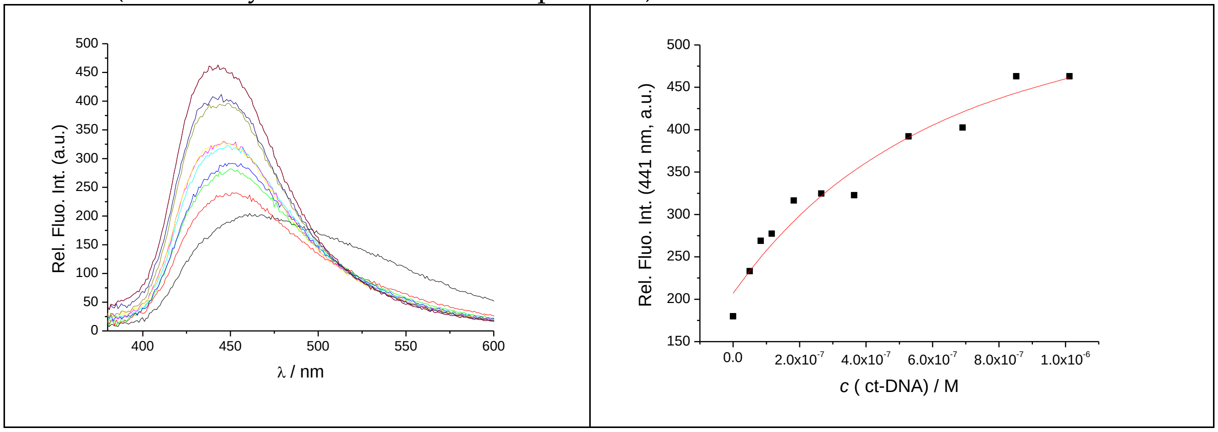

Figure S17. Left: Fluorimetric titration of **4**, λ_{exc} = 345 nm, c = 5 × 10^{-8} mol dm^{-3} with ct-DNA, Right: Experimental and calculated fluorescence intensities of **4** at λ_{em} = 441 nm upon addition of *ct*-DNA (Na-cacodylate buffer, *I*= 0.05 M, pH = 7.0)

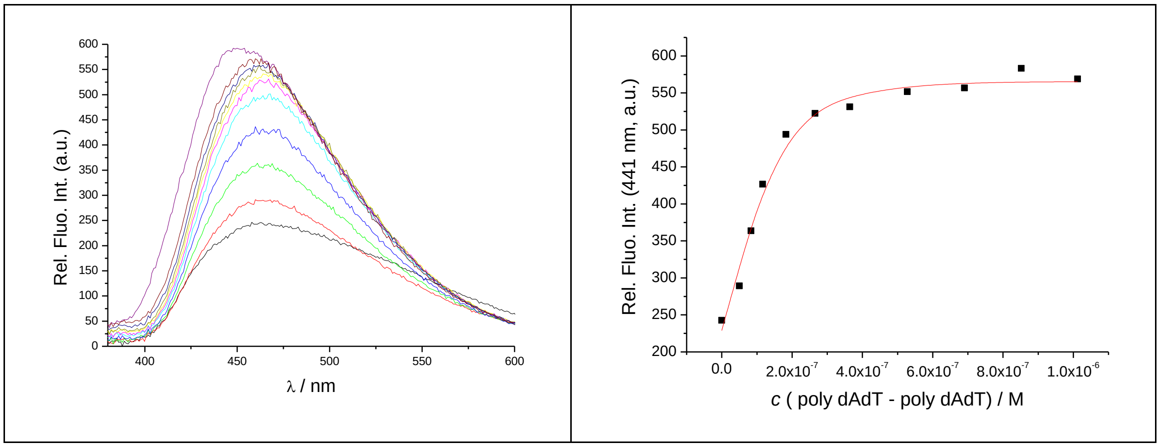

Figure S18. Left: Fluorimetric titration of **4**, λ_{exc} = 345 nm, c = 5 × 10^{-8}mol dm^{-3} with pdApdT-pdApdT, Right: Experimental and calculated fluorescence intensities of **4** at λ_{em} = 441 nm upon addition of DNA (Na-cacodylate buffer, *I*= 0.05 M, pH = 7.0).

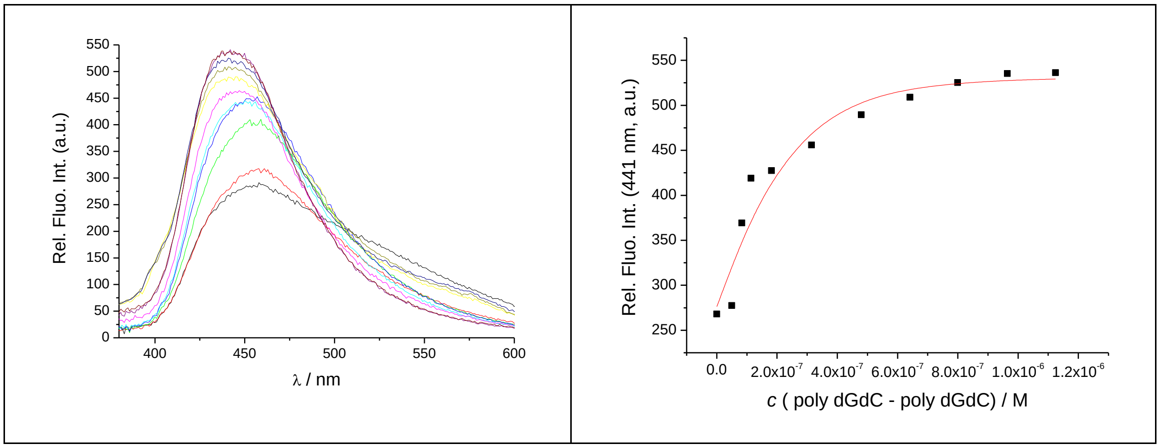

Figure S19. Left: Fluorimetric titration of **4**, λ_{exc} = 345 nm, c = 5 × 10^{-8}mol dm^{-3} with pdGpdC-pdGpdC, Right: Experimental and calculated fluorescence intensities of **4** at λ_{em} = 441 nm upon addition of DNA (Na-cacodylate buffer, *I* = 0.05 M, pH = 7.0).

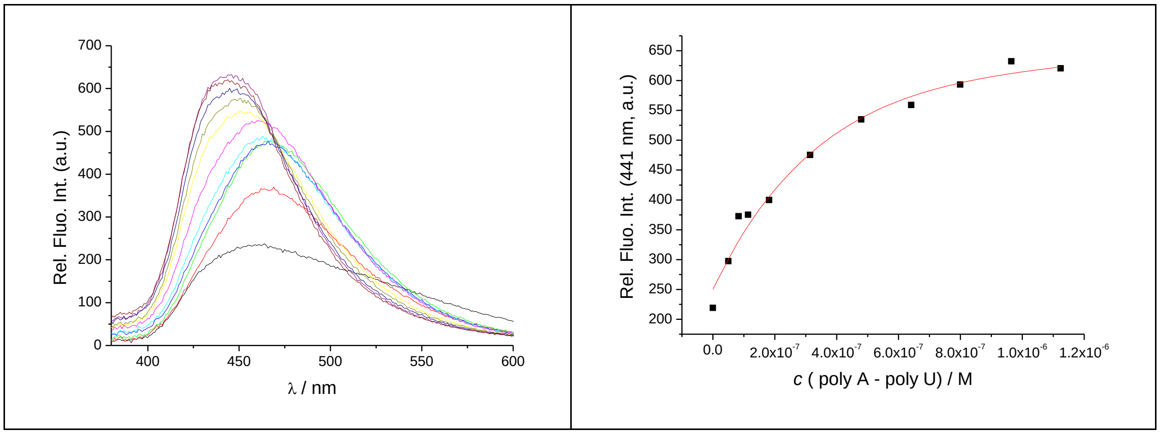

Figure S20. Left: Fluorimetric titration of **4**, λ_{exc} = 345 nm, c = 5 × 10^{-8} mol dm^{-3} with pA-pU, Right: Experimental and calculated fluorescence intensities of **4** at λ_{em} = 441 nm upon addition of DNA (Na-cacodylate buffer, *I* = 0.05 M, pH = 7.0).

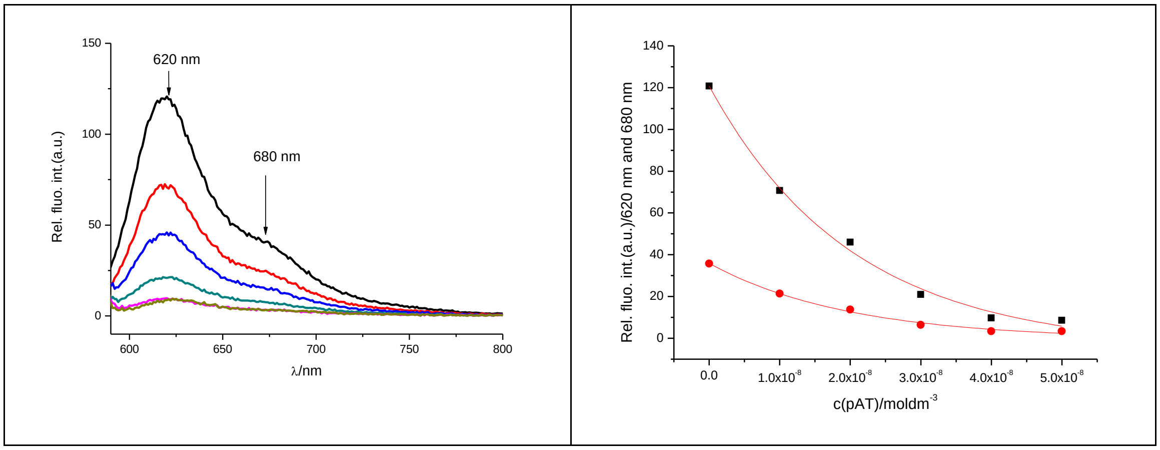

Figure S21. Left: Fluorimetric titration of **5**, λ_{exc} = 562 nm, c = 5 × 10^{-8} mol dm^{-3} with pdApdT-pdApdT, Right: Experimental and calculated fluorescence intensities of **5** at λ_{em} = 620 nm and 680 nm upon addition of DNA (Na-cacodylate buffer, *I* = 0.05 M, pH = 7.0).

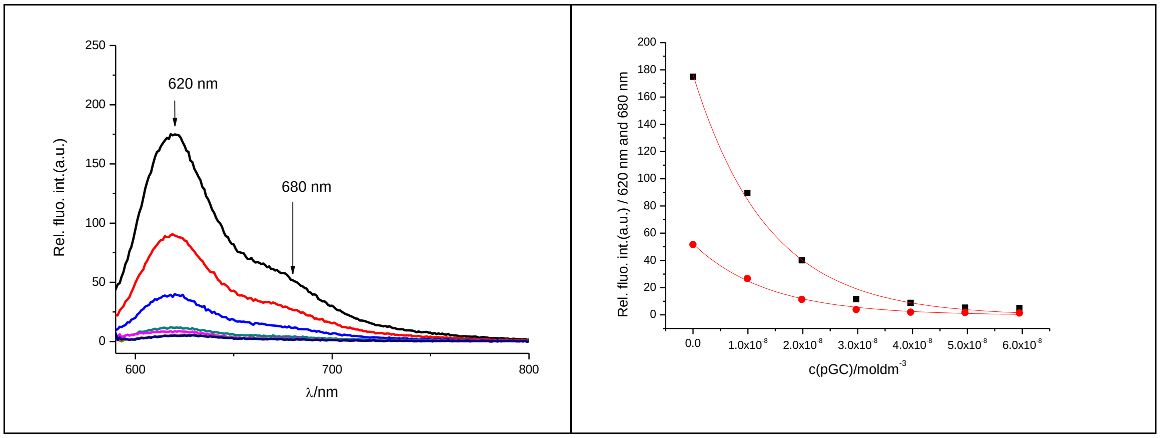

Figure S22. Left: Fluorimetric titration of **5**, λ_{exc} = 562 nm, c = 5 × 10^{-8} mol dm^{-3} with pdGpdC-pdGpdC, Right: Experimental and calculated fluorescence intensities of **5** at λ_{em} = 620 nm and 680 nm upon addition of DNA (Na-cacodylate buffer, *I* = 0.05 M, pH = 7.0).

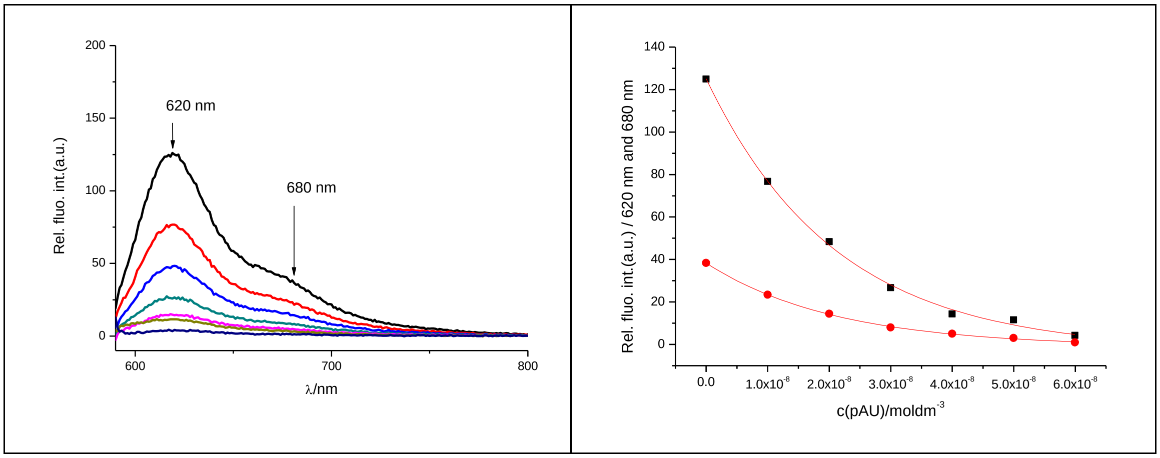

Figure S23. Left: Fluorimetric titration of **5**, λ_{exc} = 562 nm, c = 5 × 10^{-8} mol dm^{-3} with pA-pU, Right: Experimental and calculated fluorescence intensities of **5** at λ_{em} = 620 nm and 680 nm upon addition of DNA (Na-cacodylate buffer, *I* = 0.05 M, pH = 7.0).

Thermal melting experiments

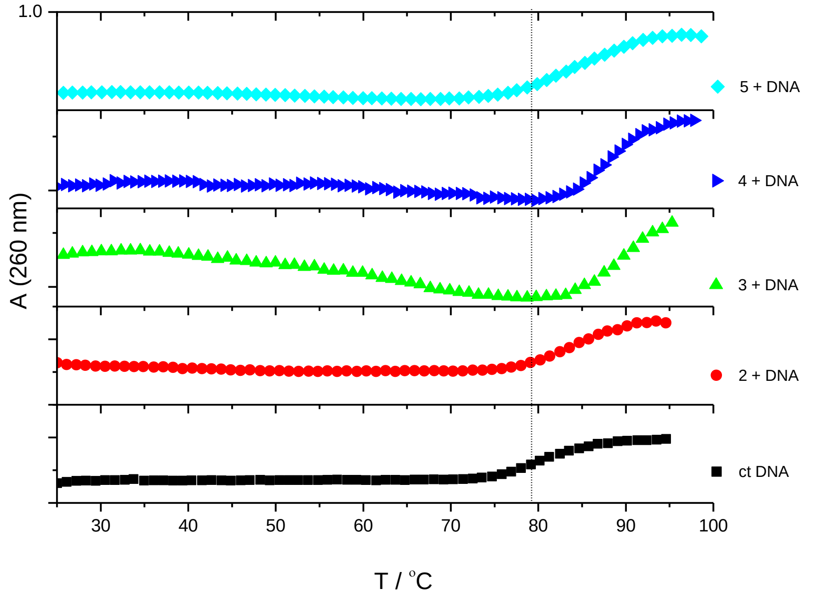

Figure S24. Melting curves of *ct* DNA upon addition of compounds **2-5** (c (DNA) = 2 ×10^{-5} M; ratio r[compound] /[polynucleotide] = 0.1) (Na-cacodylate buffer, I = 0.05 mol dm^{-3}, pH = 7.0).

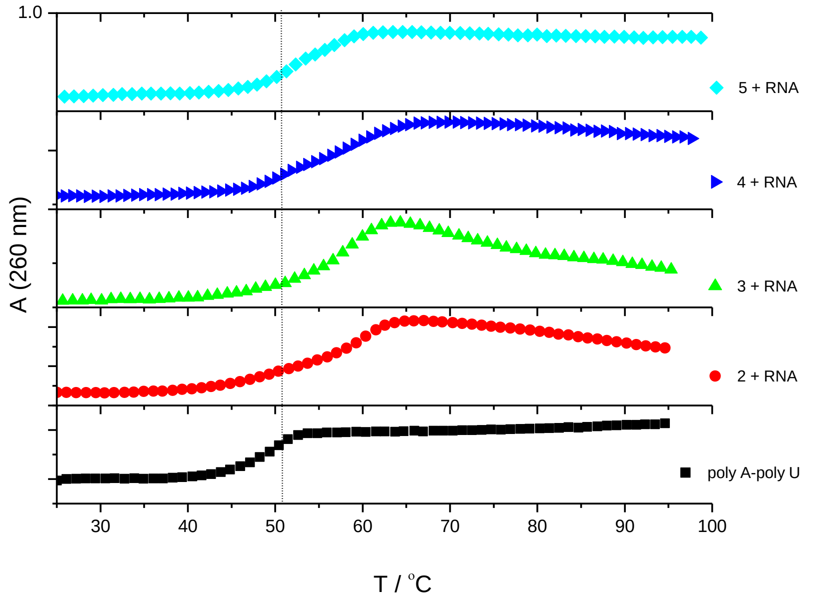

Figure S25. Melting curves of poly rA-poly rU upon addition of ompounds **2-5** (c (RNA) = 2 × 10^{-5} M; ratio r[compound] /[polynucleotide] = 0.1) (Na-cacodylate buffer, I = 0.05 mol dm^{-3}, pH = 7.0).

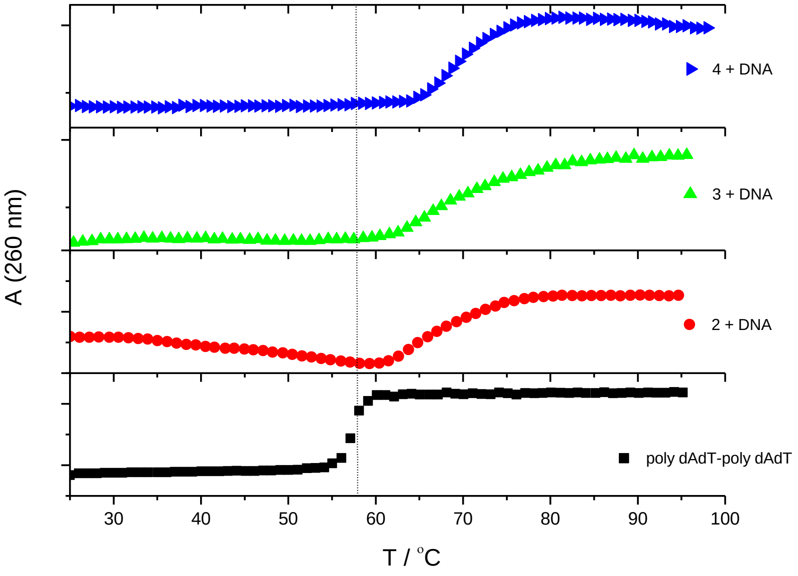

Figure S26. Melting curves of poly dAdT-poly dAdT upon addition of ompounds **2-4** (c (DNA) = 2 × 10^{-5} M; ratio r[compound] /[polynucleotide] = 0.1) (Na-cacodylate buffer, I = 0.05 mol dm^{-3}, pH = 7.0).

CD experiments

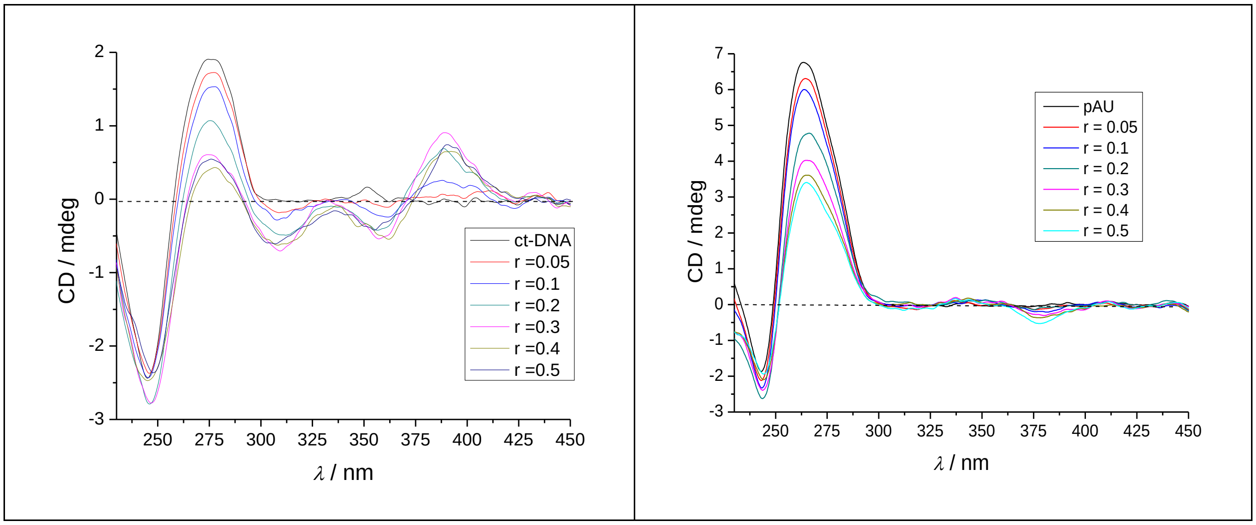

Figure S27. CD titration of ct-DNA (Left) and poly A- poly U (Right) with **2** ($c = 2 \times 10^{-5}$ M) at molar ratios ***r*** = [compound] / [polynucleotide] (pH 7.0, buffer sodium cacodylate, $I = 0.05$ M)

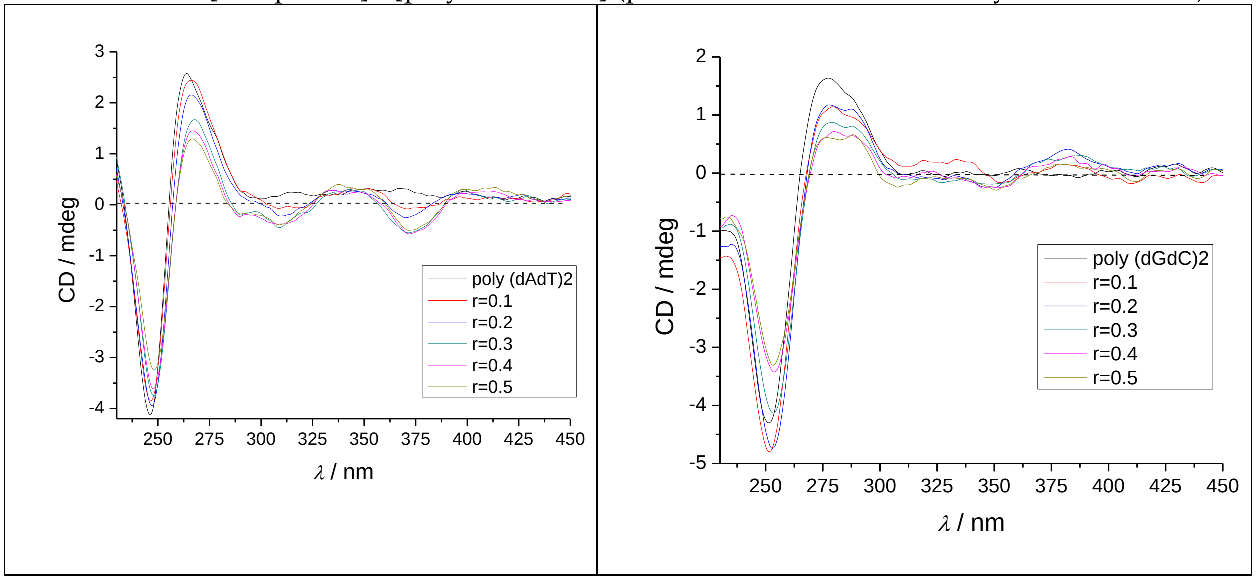

Figure S28. CD titration of poly dAdT – poly dAdT (Left) and poly dGdC - poly dGdC (Right) with **2** ($c = 2 \times 10^{-5}$ M) at molar ratios ***r*** = [compound] / [polynucleotide] (pH 7.0, buffer sodium cacodylate, $I = 0.05$ M)

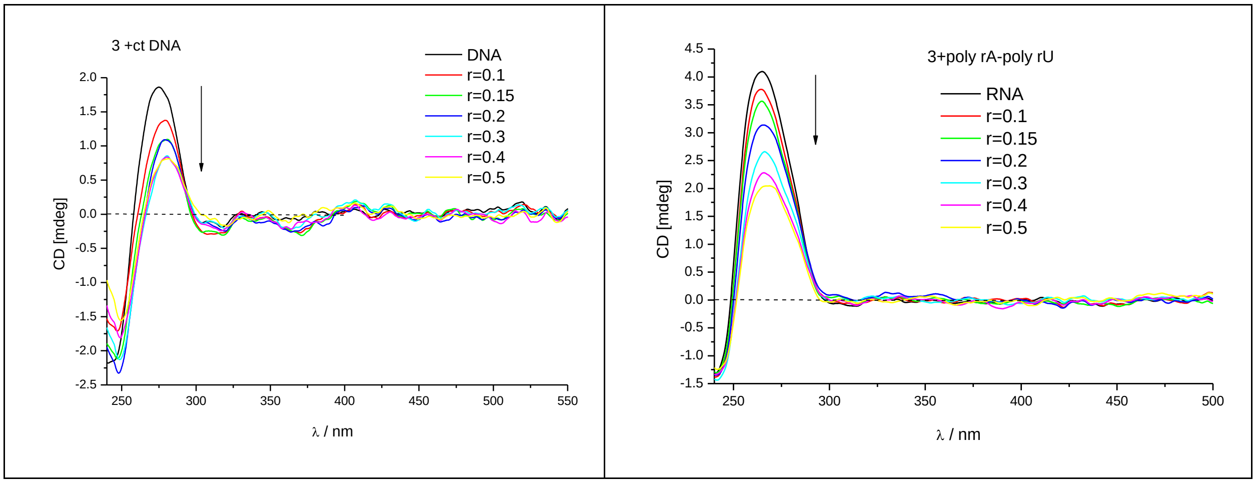

Figure S29. CD titration of ct-DNA ($c = 2 \times 10^{-5}$ M) (Left) and poly A- poly U ($c = 1.0 \times 10^{-5}$ M) (Right) with **3** at molar ratios ***r*** = [compound] / [polynucleotide] (pH 7.0, buffer sodium cacodylate, $I = 0.05$ M)

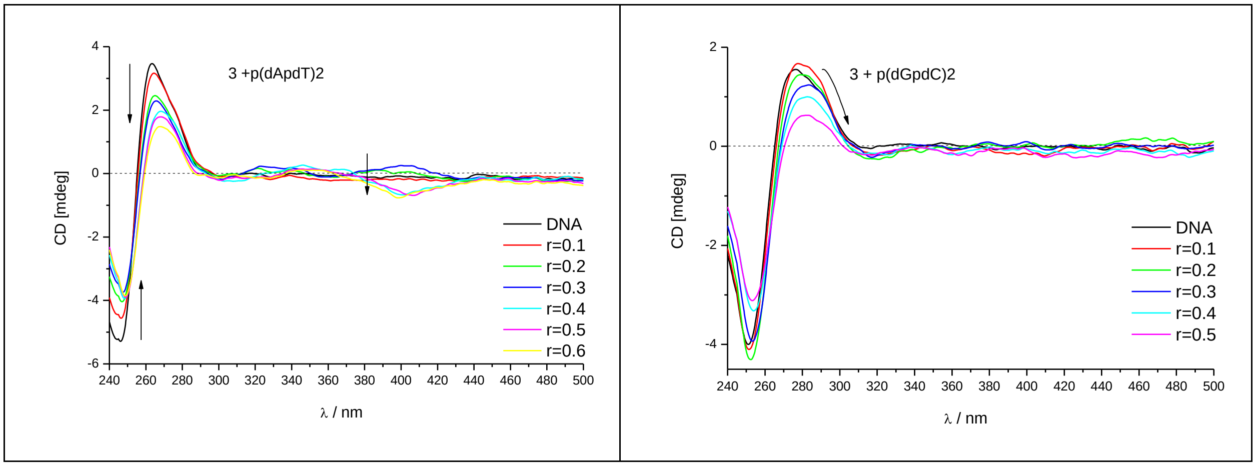

Figure S30. CD titration of poly dAdT – poly dAdT (Left) and poly dGdC - poly dGdC (Right) with **3** ($c = 2 \times 10^{-5}$ M) at molar ratios ***r*** = [compound] / [polynucleotide] (pH 7.0, buffer sodium cacodylate, $I = 0.05$ M)

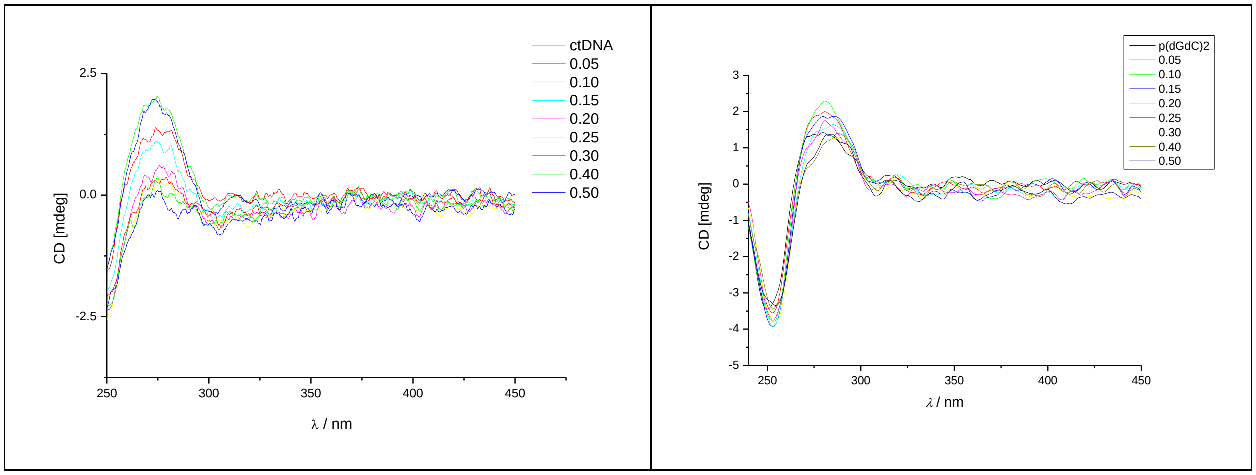

Figure S31. CD titration of ctDNA (Left) and poly dGdC - poly dGdC (Right) with **4** ($c = 2 \times 10^{-5}$ M) at molar ratios ***r*** = [compound] / [polynucleotide] (pH 7.0, buffer sodium cacodylate, $I = 0.05$ M)

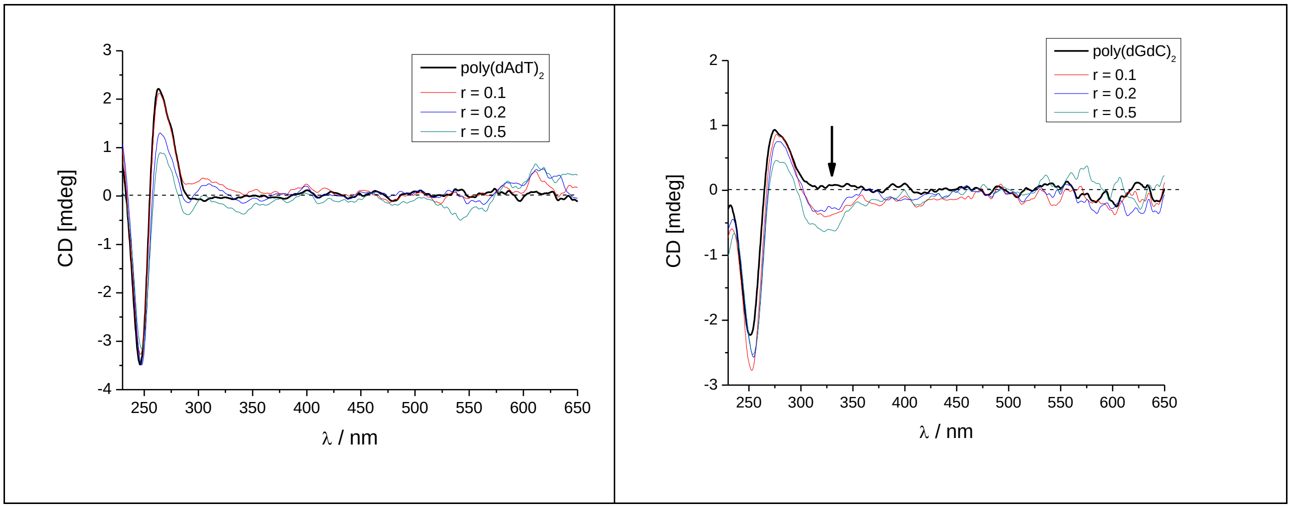

Figure S32. CD titration of poly(dAdT)$_2$ ($c = 2.0 \times 10^{-5}$ M) (Left) and poly(dGdC)$_2$ (Right) ($c = 2.0 \times 10^{-5}$ M), with **5** at molar ratios ***r*** = [**5**] / [polynucleotide] (pH 7, buffer sodium cacodylate, $I = 0.05$ M).

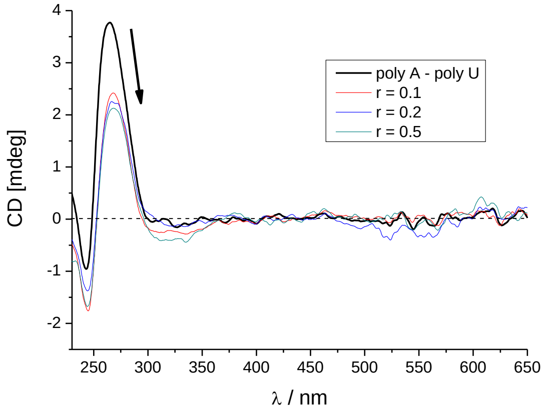

Figure S33. CD titration of poly A – poly U (c = 2.0 × 10^{-5} M), with **5** at molar ratios ***r*** = [**5**] / [polynucleotide] (pH 7, buffer sodium cacodylate, I = 0.05 M).

References

1. Saenger, W. *Principles of Nucleic Acid Structure*; Springer: New York, NY, USA, 1983; p. 226.
2. Cantor, C.R.; Schimmel, P.R. *Biophysical Chemistry Part III: The Behavior of Biological Macromolecules*; W.H. Freeman and Company: San Francisco, CA, USA, 1980; pp. 1109–1181.
3. Griesbeck, S.; Michail, E.; Wang, C.; Ogasawara, H.; Lorenzen, S.; Gerstner, L.; Zang, T.; Nitsch, J.; Sato, Y.; Bertermann, R.; et al. Tuning the π-bridge of quadrupolar triarylborane chromophores for one- and two-photon excited fluorescence imaging of lysosomes in live cells. *Chem. Sci.* **2019**, *10*, 5405–5422.
